# Elastographic Assessment of Atherosclerotic Plaques and Determination of Vascular Risk in Patients with Rheumatoid Arthritis

**DOI:** 10.3390/diagnostics14212426

**Published:** 2024-10-30

**Authors:** Velichka Popova, Stanislava Popova-Belova, Mariela Geneva-Popova, Rositsa Karalilova, Zguro Batalov, Konstantin Batalov, Mladen Doykov, Vesela Mitkova-Hristova

**Affiliations:** 1 Department of Propedeutic of Internal Diseases, Faculty of Medicine, Medical University of Plovdiv, Clinic of Rheumatology, University General Hospital “Kaspela”, 4001 Plovdiv, Bulgaria; rositsa.karalilova@mu-plovdiv.bg (R.K.); zguro.batalov@mu-plovdiv.bg (Z.B.); konstantin.batalov@mu-plovdiv.bg (K.B.); 2 Department of Propedeutic of Internal Diseases, Faculty of Medicine, Medical University of Plovdiv, Clinic of Rheumatology, University General Hospital “Sveti Georgi”, 4001 Plovdiv, Bulgaria; stanislava.popova@mu-plovdiv.bg (S.P.-B.); mariela.geneva@mu-plovdiv.bg (M.G.-P.); 3 Department of Urology and General Medicine, Medical Faculty, Medical University of Plovdiv, Bulgaria Clinic of Urology, University General Hospital “Kaspela”, 4001 Plovdiv, Bulgaria; mladen.doykov@mu-plovdiv.bg; 4 Department of Ophthalmology, Faculty of Medicine, Medical University of Plovdiv, University Clinic of Ophthalmology, University General Hospital, “Sveti Georgi”, 4001 Plovdiv, Bulgaria; vesela.hristova@mu-plovdiv.bg

**Keywords:** shear wave elastography (SWE), two-dimensional shear wave elastography (QElaXto 2D), rheumatoid arthritis (RA), atherosclerotic plaques, vascular risk

## Abstract

**Objectives**: The present study aimed to examine the role of two-dimensional shear wave elastography (SWE) in the assessment of the vascular wall of the carotid arteries and atherosclerotic plaques in patients with rheumatoid arthritis with moderate and low disease activity versus healthy controls. **Methods**: An observational case–control study was carried out at the University Medical Hospital “Kaspela” in Plovdiv, Bulgaria, from June 2023 to August 2024. This study included 24 patients with rheumatoid arthritis (RA) and 25 healthy controls. We employed two-dimensional SWE (2D-SWE) to examine the vessels around the plaques. The potential links with the degree of stenosis, plaque type, and cardiovascular risk were analyzed. **Results**: In the RA group, the 2D-SWE values showed significant positive correlations with the severity of the atherosclerotic plaques (rs = 0.461; 95% CI: 0.049 to 0.739; *p* = 0.023) and the degree of stenosis (rs = 0.920; 95% CI: 0.793 to 0.970; *p* < 0.001). Based on 2D-SWE, a ROC curve analysis distinguished higher severity plaques from lower severity plaques with an AUC = 0.818, 95% CI: 0.683 to 0.913. The optimal cut-off value of 2D-SWE > 32.40 kPa was associated with a sensitivity of 96%, a specificity of 56%, a positive predictive value (PPV) of 66.70%, and a negative predictive value (NPV) of 92.90%. **Conclusion:** Elastography can be an effective technique for assessing and stratifying atherosclerotic plaques in patients with RA, as well as for aiding in the early detection and subsequent prevention of future complications.

## 1. Introduction

Elastography is a medical technique that evaluates the elasticity of tissue by measuring its capacity to resist deformation by an applied force and regain its original shape after the pressure is removed [1]. Shear wave elastography (SWE) generates transverse waves that propagate through the tissue structure under examination using a high-power acoustic radiofrequency [2]. In shear wave ultrasound (US) imaging, the shear wave velocity is linked to the shear modulus, and particle motion is perpendicular to the direction of wave propagation [1,3]. It is applied in a variety of medical conditions in which deviations in tissue elasticity are associated with pathological processes [4]. However, it is mostly used in oncology and gastroenterology [5,6] because, in some medical conditions, there is an absence of standardized reference values of normal tissue elasticity.

The liver, the spleen, the pancreas, the salivary glands, the prostate, and the mammary and thyroid glands are among the recent diagnostic applications of SWE [5,7]. It is increasingly being employed in musculoskeletal sonography [8], as well as in dermatology and rheumatology, particularly to evaluate the skin of patients with progressive systemic sclerosis [6]. The first uses of SWE in rheumatology were to assess the Achilles tendon [9], skin density, and subcutaneous tissue in scleroderma [10].

In 2014, the SWE methodology was utilized for the first time to measure the density and vulnerability of plaques by quantifying the Young’s modulus [8]. The assessment of qualitative changes in the structure of carotid vessels and different types of atheromatous plaques can help in the calculation of vascular risk in patients with serious comorbid conditions [11]. In rheumatology, the early onset of vascular accidents in patients with inflammatory and autoimmune diseases necessitates this assessment, and it could serve as a methodology for examining the vessel walls in vasculitis. SWE is widely applied to assess vascular changes incases with rheumatoid arthritis, where the risk of early cardiovascular (CV) death and cerebrovascular disease (CVD) occurs 10 years earlier compared to the rest of the population [12]. As a non-invasive and accessible technique for the stratification of patients, SWE can assist in the identification of patients at risk and the prevention of potential incidents by implementing appropriate therapy [13].

Atherosclerotic plaques are rarely homogeneous; in most cases, they are heterogeneous with calcifications, erosions, and hemorrhages [14]. Intraplaque hemorrhages are the main substrate and cause of rupture, but their visualization is difficult with standard sonography [15]. The presence of risk or defect in the plaques may only be ascertained using invasive techniques and MRI [16,17].

The most recent EULAR recommendations stipulate that the carotid intima media ratio (cIMT) should be used to evaluate the vessels. However, cIMT is associated with a number of drawbacks, including the fact that it may vary with age, it does not assess plaque morphology because it is conducted in an area outside the presence of plaque, it is not always associated with a real risk, and there are conflicting findings regarding the relationship between obesity and the risk of cardiovascular (CV) events [18]. Rheumatoid arthritis is the most extensively researched inflammatory joint disease and serves as a standard for cardiovascular risk evaluation in rheumatology. Systemic inflammation is a critical pathogenic contributor to the early onset of complex atherosclerosis and the formation of atherosclerotic plaques of types 4 and 5 [10].

Elastography has the potential to serve as an appropriate alternative for evaluation due to its ability to quantitatively and qualitatively assess plaques [19]. SWE can be used as a routine methodology for studying the quality of vascular walls [20], as well as for determining the pathological and age-related changes in the elasticity of arterial vessels. Furthermore, it can be introduced as a diagnostic method for evaluating the vascular wall in vasculitis that affects large blood vessels, like M. Horton’s and Takayasu’s arteritis.

Research on the vascular wall has so far involved small patient groups, thus not allowing the standardization of the methodology [21]. Nevertheless, SWE, in conjunction with strain wave elastography, is increasingly utilized for the endovascular evaluation of atherosclerotic plaques and their vulnerability due to its non-invasiveness and accessibility [22]. We believe that the two methods are mutually beneficial, with each having its own advantages and disadvantages. Shear wave elastography is generally considered more suitable for vascular wall assessment due to the fact that it does not require pressure, whereas strain wave elastography does [22,23].

The present study aimed to examine the role of two-dimensional SWE in the assessment of the vascular wall of the carotid arteries and atherosclerotic plaques in patients with rheumatoid arthritis with moderate and low disease activity versus healthy controls. Specifically, we examined the relationship between plaque type, degree of stenosis, elasticity (kPa), and future vascular risk. Criterion values of 2D-SWE were determined to differentiate between severe atherosclerotic plaques (types 3, 4, and 5) and moderate ones (types 1 and 2)

## 2. Materials and Methods

### 2.1. Study Design

An observational case-control study was carried out at the University Medical Hospital “Kaspela” in Plovdiv, Bulgaria, from June 2023 to August 2024. The research protocol was reviewed and approved by the committee of scientific ethics at the University Hospital “Kaspela” (approval code No. 17/1 June 2023). All procedures were in compliance with the requirements of the World Medical Association’s Declaration of Helsinki (1964) and its 2000 revision (Edinburgh). All participants provided written informed consent for using the data in scientific publications.

### 2.2. Participants

This study included 24 patients (17 women and 7 men) who had been diagnosed with rheumatoid arthritis for 10 years or more and had mild to moderate disease activity, as determined by DAS28. The disease condition of the patients was managed through the administration of appropriate therapies. Eleven patients were treated with TNF-alpha inhibitors, seven of whom received adalimumab at a dosage of 0.040 subcutaneously every 14 days, while the other four received etanercept at a dosage of 0.050 once weekly. Five of the patients who were taking TNF-α inhibitors were concurrently receiving methotrexate 10–15 mg weekly, and six were receiving leflunomide × 1 t. of 0.020 daily. Eight individuals received treatment with JAK inhibitors, including four on 15 mg rinvog and four on 11 mg tofacitinib. Five individuals received IL6 inhibitors, specifically 162 mg RoActemra once weekly.

A control group of 25 clinically healthy patients (18 women and 7 men) was also included. Within each group, we formed subgroups based on the morphology of atherosclerotic plaques: (1) a low-risk group, including atherosclerotic plaques of types 1 and 2 according to the Gray–Weale classification, and (2) a group of high-risk patients with atherosclerotic plaques of types 3 to 5 [24].

The inclusion criteria for the patients with RA were (1) a confirmed diagnosis based on the EULAR/ACR 2010 criteria; (2) evidence of disease activity with DAS28, where values greater than or equal to 5.12 show high disease activity, values between 2.6 and 5.1 show moderate disease activity, and values less than or equal to 2.6 show low disease activity.

Patients were excluded from participation in this study in the following cases: (1) intake of lipid-lowering therapy; (2) intake of systemic glucocorticoids (GCSs), as well as locally administered; (3) a vascular accident or coronary revascularization; (4) an acute inflammatory condition.

### 2.3. Procedure

We used Esaote MylabX8 device (Esaote Esaote S.p.A.-sole-shareholder company Via Enrico Melen 77, 16152 Genova, Italy) to perform two-dimensional shear wave elastography (QElaXto 2D) on the vessels around the plaque. In 2D-SWE, acoustic radiation is used from multiple focal zones that propagates in very rapid succession, faster than the shear wave’s speed. This enables the measurement of shear wave velocity, or Young’s modulus, and the generation of color quantitative elastograms in real time [25]. The examination of the vessels was carried out at the time of diastole and during breath hold, with the control histogram in the green range, with repetition cycles lasting 3–10 s. All measurements were made by two independent researchers.

The following parameters were evaluated for all patients: the grade of stenosis as determined by the European Carotid Surgery Trial (ECST), the future 10-year risk of cardiovascular disease as determined by the Systematic Coronary Risk Evaluation 2-Older Persons (SCORE2-OP), disease activity as determined by the DAS28, the type of plaques as determined by the Gray–Weale classification, and the plaque elasticity as determined by the QElaXto 2D.

Plaques and vessel walls were evaluated by SWE, measured in kPa in the area of interest. Three to ten measurements were made and standardized. Color plaque images were assessed versus histograms for qualitative assessment and quantitative measurement. By quantifying the mechanical and elastic properties of the tissues, SWE complemented the diagnosis obtained in the sonographic examination in B-mode and power and color Doppler.

The carotid arteries were examined by two physicians using a multi-frequency linear transducer (4–15 MHz) with a frequency of 10 MHz on an Esaote MylabX8 device (Esaote Esaote S.p.A. - sole-shareholder company Via Enrico Melen 77, 16152 Genova, Italy) in B-mode. The area of interest in QElaXto 2D was indicated with a 0.2 mm circle of a correct zone if one was available. A trace was used to designate the area of interest if the standard scan was not accurate and the area was not particularly large or circular. This comprised the common carotid artery, its bifurcation, and the onset of the internal and external carotid arteries.

**Figure 1:**
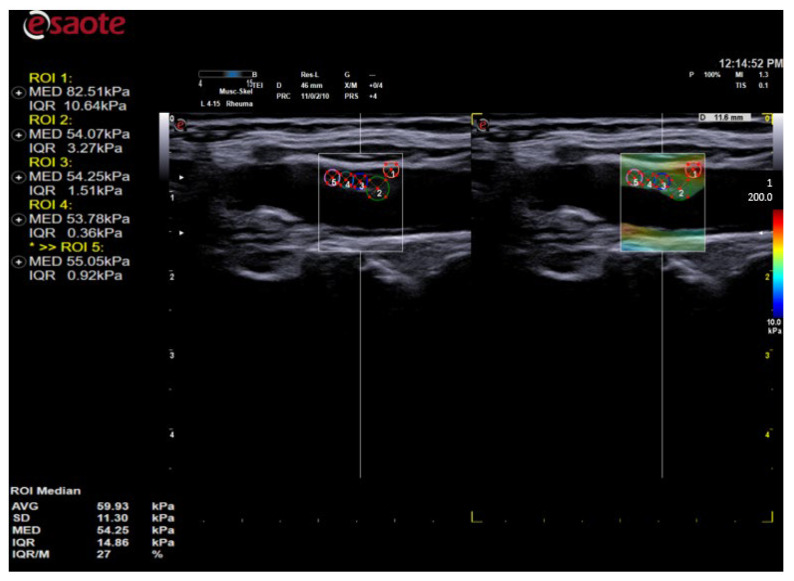
QElaXto 2D elastogram of a patient with rheumatoid arthritis and atherosclerotic plaque covering the longitudinal proximal wall of a. carotis communis near the bulbus caroticus before bifurcation.

The evaluation of the vessel wall was in the diastole phase so that there was no change in the vessel wall during systole in the apneic state. The settings of the device were as follows (Figure 1):

∘ Maximum acoustic power;
∘ About 60–75% gain;
∘ Off persistence;
∘ The area of interest and the standard scan of the bifurcation against the Mannheim consensus;
∘ The maximum stiffness of the elastogram correlating with the red color and values up to 200 kPa;
∘ The minimum value being that of blood, which was used to compare the soft structures in blue color (0 kPa);
∘ Frequency of SWE frames at 1 Hz;
∘ Processing power and frames per second: 10 frames per second.

The plaques were evaluated for their morphology and surface:

∘ Morphology: It was evaluated using real-time B scanning or duplex scanning, taking into account characteristics such as echogenicity (density), which distinguishes the following types of changes: (1) anechoic plaques—standardized to blood; (2) isoechoic—standardized, relative to the mastoid muscle; (3) hyperechoic—standardized, relative to the bone.
∘ Surface: The classification included (1) smooth (regular)—the cIMT complex is continuous and without irregularities; (2) uneven (irregular)—cIMT is interrupted and uneven; (3) hollow—showing more than 2 mm of concavity; (4) ulcerated—the surface is uneven, hollow, with an effusion of lipid substances and blood [26,27].

For the purpose of our investigation, we categorized the plaques into 5 types, following the Gray–Weale classification. [25]:

∘ Type 1: Plaques of this type are uniformly echolucent (black), with less than 15% of the plaque area occupied by colored areas, i.e., no more than 25 pixels from the gray scale. If the fibrous cap is not visible, the plaque can only be detected using color, power Doppler, and B flow.
∘ Type 2: Mainly echotransparent, with the colored areas occupying 15% to 50% of the area of the plaque.
∘ Type 3: Mainly echogenic, with colored areas occupying 50% to 85% of the plaque area.
∘ Type 4 and 5: Uniformly echogenic, with stained areas occupying more than 85% of the plaque area.

Sometimes, the distinction between lesions 4 and 5 can only be made histologically, which is why they are often referred to as types 4 and 5 fibrous plaques. Type 4 atherosclerotic plaques are lesions characterized by a dense accumulation of extracellular lipids, which act as a lipid core and occupy an extensive, over 85%, but well-defined intimal area. The degranulation of the intima and accumulation of fibrous tissue can cause lesion defects, rupture, and thrombosis. The process of changing the lipid core and increasing the fibrous tissue turns the plaque into type 5 [25].

The assessment of CV risk was based on the SCORE 2-OR scale, calibrated for individual regions, according to which patients were divided into (1) high risk (>10%) in the presence of two or more risk factors; (2) moderately high risk (5–10%) in the presence of two or more risk factors; (3) moderate risk (1–5%) when two or more risk factors were present; (4) low risk (<1%) when none or only one risk factor was present. Other risk factors included age, sex, smoking, total cholesterol, HDL cholesterol, and systolic arterial pressure [28].

### 2.4. Statistical Methods

The data were analyzed through Statistical Software for the Social Sciences (SPSS) Version 27 (2020). The Shapiro–Wilk test was used to screen the continuous variables for normality. For normally distributed variables, the central tendency is described with means and standard deviations (SDs), and between-group comparisons were performed through independent-samples *t*-tests. In the absence of normality, the medians and interquartile ranges (IQRs) were calculated, and the Mann–Whitney test was used for comparing the study groups. The receiver operating characteristic (ROC) curve was employed to establish the diagnostic potential of 2D-SWE in distinguishing severe plaques (types 3, 4, and 5) from mild ones (types 1 and 2). Categorical variables are summarized by frequencies and percentages, and associations were established through the chi-square test and Fisher’s exact test. Spearman’s rank-order correlation was determined to examine the relationship between target variables. All statistical tests were two-tailed and performed at a Type I error (α) of 0.05.

## 3. Results

There was no significant age difference between the patients with RA and the healthy controls (*p* = 0.813). In both groups, the women constituted the bigger proportion: 70.80% of the RA group and 73.10% of the healthy controls (*p* = 1.000). There was no significant difference related to the BMI (*p* = 0.671) and smoking (*p* = 0.373) risk factors. Significant differences were found between the groups in the degree of stenosis (*p* < 0.0001) and the types of atherosclerotic plaques (*p* < 0.001). In the RA group, there was a prevalence of the more severe stages compared to the complete absence of these in the healthy controls. For the patients with RA, DAS28 had a mean of 2.98 ± 0.60, with a minimum score of 2.10 and a maximum of 4.54 (Table 1).

### 3.1. Two-Dimensional Point Shear Wave Elastography (2D-SWE) of Atherosclerotic Plaques in Patients with RA and Healthy Controls

**Figure 2:**
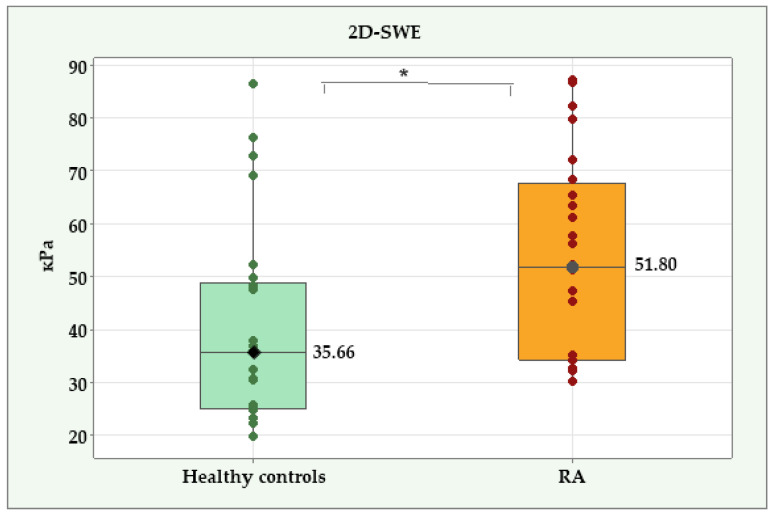
Two-dimensional point shear wave elastography (2D-SWE) in patients with RA and healthy controls. *—Significant difference at *p* < 0.05.

The 2D-SWE of the atherosclerotic plaques showed significant differences between the patients with RA and the healthy controls (Figure 2). The median 2D-SWE had a value of 51.80 kPa (IQR = 33.52) in the RA group versus 35.66 kPa (IQR = 23.65) in the healthy controls (*p* = 0.011).

**Figure 3:**
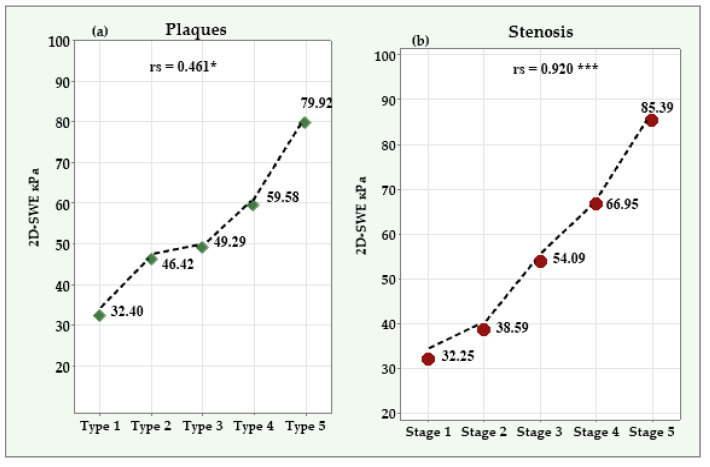
Significant positive correlations between 2D-SWE values, the severity of atherosclerotic plaques (panel **a**), and the degree of stenosis (panel **b**) in patients with RA. *—Significant correlation at *p* < 0.05; ***—significant correlation at *p* < 0.001.

In the RA group, the 2D-SWE values showed significant positive correlations with the severity of the atherosclerotic plaques (r^s^ = 0.461; 95% CI: 0.049 to 0.739; *p* = 0.023) and the degree of stenosis (r^s^ = 0.920; 95% CI: 0.793 to 0.970; *p* < 0.001) (Figure 3). No significant association was found between the 2D-SWE values and DAS28 (r^s^ = 0.347; 95% CI: −0.078 to 0.665; *p* = 0.096). In the healthy control group, no significant associations were found between the 2D-SWE values, the severity of the atherosclerotic plaques (r^s^ = 0.169; 95% CI: −0.237 to 0.524; *p* = 0.410), and the degree of stenosis (r^s^ = 0.221; 95% CI: −0.187 to 0.563; *p* = 0.279).

### 3.2. Evaluation of the Diagnostic Capability of 2D-SWE in Differentiating Between Severe (Types 3, 4, and 5) and Moderate (Types 1 and 2) Atherosclerotic Plaques

**Figure 4:**
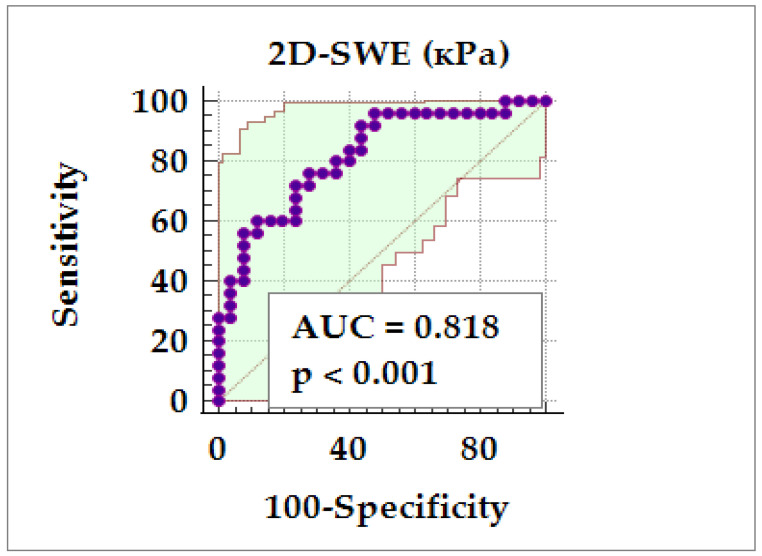
Receiver operating characteristic curve for the diagnostic potential of 2D-SWE in distinguishing severe (types 3, 4, and 5) from less severe (types 1 and 2) atherosclerotic plaques.

We performed a ROC curve analysis, coding all patients with RA with more severe atherosclerotic plaques (types 3, 4, and 5) as group 1, and all patients with RA with mild atherosclerotic plaques (types 1 and 2) as group 0. The results showed a significant area under the curve (AUC = 0.818; 95% CI: 0.683 to 0.913; *p* < 0.001). The optimal cut-off value of 2D-SWE > 32.40 kPa was associated with a sensitivity of 96%, a specificity of 56%, a positive predictive value (PPV) of 66.70%, and a negative predictive value (NPV) of 92.90% (Figure 4).

### 3.3. SCORE2-OP in Patients with RA and Healthy Controls

**Figure 5:**
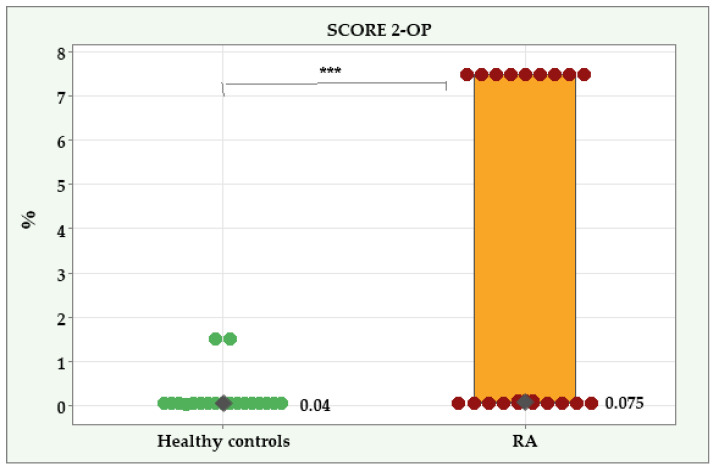
SCORE 2-OP in patients with RA and healthy controls. ***—Significant difference at *p* < 0.001.

The SCORE2-OP had a significantly higher median value in the RA group (0.075%; IQR = 7.45) compared to the healthy controls (0.04%; IQR = 0.01), *p* < 0.001 (Figure 5). No significant differences were found between the men and women in the RA group (*p* = 0.349) or in the healthy controls (*p* = 0.910).

We found a significant positive correlation between 2D-SWE and the SCORE 2-OP in the RA group (r^s^ = 0.557; 95% CI: 0.164 to 0.796; *p* = 0.005). In the healthy control group, no significant association existed between the two variables (r^s^ = 0.216; 95% CI:—0.192 to 0.560; *p* = 0.290).

The SCORE 2-OP was not significantly associated with the severity of atherosclerotic plaques in the RA group (r^s^ = 0.191; 95% CI: −0.234 to 0.555; *p* = 0.371) or in the healthy controls (r^s^ = 0.286; 95% CI: −0.128 to 0.607; *p* = 0.165).

## 4. Discussion

Atherosclerotic plaques are rarely homogeneous; often, they are heterogeneous with calcifications, lipid accumulations, erosions, and hemorrhages. Erosions, the presence of intraplaque hemorrhages, and the rupture of the fibrous cap contribute to their instability and rupture risk, all of which are challenging to identify using conventional methods. Soft plaques, as well as those with pronounced densities of types 4 and 5, are significantly more likely to rupture and pose a serious risk to patients’ lives [14,15,16,17].

Not only patients with inflammatory joint diseases but also a large part of the elderly population are at high risk. Asymptomatic lesions persist for a long time until the plaque ruptures and its contents embolize, leading to a subsequent vascular accident. Elastography primarily serves to evaluate parenchymal organs, distinguish conditions associated with chronic liver damage leading to liver fibrosis or cirrhosis, and distinguish benign from malignant lesions in oncology [5,6].

In rheumatology, vascular assessment is still in its infancy and somewhat underexplored. In addition, there is a lack of standardized methods, criterion values, and sufficient data to determine the risk groups for plaque rupture. Our results showed that plaques of types 3, 4, and 5 predominated in patients with rheumatoid arthritis. In the latter group, 2D-SWE (kPa) showed a positive correlation with plaque severity and degree of stenosis. In comparison, 2D-SWE had significantly lower values in the healthy controls and was not significantly associated with the severity of plaques or the degree of stenosis.

The compaction of the vascular wall results in a change in its elasticity, which in turn creates conditions for intraplaque hemorrhage or rupture, leading to an effusion of its contents and subsequent thrombosis and vascular accidents. As a result, patients with rheumatoid arthritis have a higher degree of stenosis, as well as a higher density and a higher heterogeneity of plaques, which defines them as being at higher risk and with a higher frequency of vascular incidents [13,14].

The assessment of plaques and the consequences associated with the change in their morphology have led to the creation of new research techniques such as 3D assessment and the advent of elastography. These developments provide new opportunities for early risk assessment for these patients [28]. The vascular wall’s thickening and age-related changes should be considered; however, standardized methodologies are still necessary in this context [8,29,30].

The equivalent elasticity images are calculated from the estimation of the vascular tissue movement, but the parameters are difficult to estimate because the tissue movement from the blood flow is radial in the vessel wall, while the ultrasound beam propagates axially, which necessitates the use of different evaluation methodologies [8].

Another unexplored area in rheumatology is the use of elastography to assess the vascular changes in large vessel vasculitis. In these cases, elastography could play a crucial role in identifying qualitative changes. Unfortunately, to the best of our knowledge, there are no related studies on patients with inflammatory joint diseases or vasculitis. Acute vascular events appear earlier in these patients, which are atypical and more severe, depending on specific cardiovascular factors [11]. In our study, we made a positive step in this direction by establishing that, based on 2D-SWE values (kPa), plaques of types 3 and 4 could be distinguished from plaques of types 1 and 2 with 81.80% accuracy at an optimal cut-off value of 2D-SWE > 32.40 kPa. We also observed a positive correlation between 2D-SWE and the SCORE2-OP in the RA group, which was absent in the healthy controls. These findings can be used in clinical practice to identify patients with an increased risk of cardiovascular events.

Maurice et al. conducted a non-invasive assessment of the carotid vessels and found a variation in the vessel’s elasticity between the male and female sexes (33–106 kPa for the male sex and 25–67 kPa for the female sex), as well as a significant differences associated with age groups [31]. According to other researchers, it is not the degree of stenosis but the type of plaques and the presence of a fibrous calcified cap with heterogeneous contents that determine the higher risk of rupture [27]. 

Although we did not investigate sex differences in relation to SWE, it should be mentioned that the majority of our patients (70.80%) were women aged 50 and above. Substantial research has linked menopausal women to an increased risk of atherosclerotic vascular changes. Hormonal imbalances, particularly those associated with estradiol, hypercholesterolemia, and metabolic syndrome, along with the systemic proinflammatory response in rheumatoid arthritis, significantly increase the risk of cardiovascular events [32]. From this perspective, we can use SWE to compare pre- and post-menopausal patients with RA and study the impact of menopause on plaque composition.

### Limitations

The results of this study are subject to several limitations, one of which relates to the small sample size and the constraints it imposes on the possibility of examining different subgroups of patients based on demographic and clinical factors. The inability to verify our results against others due to the insufficient research on this issue is another limitation to their generalizability.

Intraplaque hemorrhage, demonstrated by the appearance of a soft density within it and a decrease in the level in kPa, possibly localized patchy areas within the plaques, would be reasons for suspicion for the risk of rupture. An important limitation is the size of the circle, a limit of 0.2 mm, which makes measurement accuracy difficult to achieve. In practice, studies classifying patients according to risk would be useful, but achieving cut-off values to define high- and low-risk patients requires larger study cohorts. Modern recommendations would incorporate their results into daily practice.

The shear modulus is sensitive to probe positioning, and shear waves are able to propagate faster along the longitudinal axes of structures. On the other hand, the shear modulus is directly related to Young’s modulus of elasticity. There are also difficulties that arise due to the combination of various factors such as the habits of the patient, the homogeneity of the tissues and overlying structures, and the access to the anatomical area [33].

## 5. Conclusions

Elastography is a promising technique for assessing the quality of the atherosclerotic plaques and vessel walls in adult patients with a variety of diseases. Our results show that it can be used in the assessment and stratification of atherosclerotic plaques in patients with rheumatoid arthritis. Moreover, elastography can help in the early detection of high-risk patients and the subsequent prevention of future complications. The current study demonstrates the potential of this technique in clinical trials and in our daily practice for qualitative judgments, diagnosis, and disease condition management.

The SWE methodology, which is still in development, is better suited for evaluating large vessels because the device’s factory settings determine the dimensions of the examined region. However, it has real potential for assessing smaller vessels, which could prove beneficial in fields like sports medicine and pediatrics.

## Figures and Tables

**Table 1:** Background information.

Variables	RA Group (*n* = 24)	Healthy Controls (*n* = 26)	*p*-Value
Age			
∘ Mean (SD)	56.17 (7.07)	56.62 (6.26)	0.813 t
Sex, *n* (%)			
∘ Women	17 (70.80%)	19 (73.10%)	
∘ Men	7 (29.20%)	7 (26.90%)	1.000 f
BMI			
∘ Mean (SD)	29.83 (3.90)	29.25 (5.46)	0.671 t
Smoking, *n* (%)			
∘ Yes	10 (41.70%)	7 (26.90%)	0.373 f
∘ No	14 (58.30%)	19 (73.10%)	
Degree of stenosis, *n* (%)			<0.001 χ2
∘ Stage 1	5 (20.80%)	14 (53.80%)	
∘ Stage 2	4 (16.70%)	12 (46.20%)	
∘ Stage 3	7 (29.20%)	0 (7.70%)	
∘ Stage 4	5 (20.80%)	0 (7.70%)	
∘ Stage 5	3 (12.50%)	0 (3.80%)	
Atherosclerotic plaques, *n* (%)			<0.001 χ2
∘ Type 1	1 (4.20%)	16 (61.50%)	
∘ Type 2	5 (20.80%)	12 (38.50%)	
∘ Type 3	7 (29.20%)	0 (0.00%)	
∘ Type 4	10 (41.70%)	0 (0.00%)	
∘ Type 5	1 (4.20%)	0 (0.00%)	
DAS28			
∘ Mean (SD)	2.98 (0.60)	N/A	N/A

t—independent-samples *t*-test; f—Fisher’s exact test; χ2—chi-square test; N/A—not applicable.

## Data Availability

The data are available from the corresponding author upon reasonable request.
